# FGF1 alleviates LPS-induced acute lung injury via suppression of inflammation and oxidative stress

**DOI:** 10.1186/s10020-022-00502-8

**Published:** 2022-06-28

**Authors:** Qhaweni Dhlamini, Wei Wang, Guifeng Feng, Aiping Chen, Lei Chong, Xue Li, Quan Li, Jin Wu, Depu Zhou, Jie Wang, Hailin Zhang, Jin-San Zhang

**Affiliations:** 1 International Collaborative Center on Growth Factor Research, School of Pharmaceutical Sciences, Wenzhou Medical University, Wenzhou, Zhejiang 325035 China; 2 Department of Pediatric Respiratory Medicine, National Key Clinical Specialty of Pediatric Respiratory Medicine, The Second Affiliated Hospital and Yuying Children’s Hospital of Wenzhou Medical University, Wenzhou, Zhejiang 325027 China; 3 College of Life and Environmental Science, Wenzhou University, Wenzhou, Zhejiang 325035 China; 4 Department of Endocrinology, The Second Affiliated Hospital of Wenzhou Medical University, Wenzhou, Zhejiang 325027 China; 5 Department of Pulmonary and Critical Care Medicine, The First Affiliated Hospital of Wenzhou Medical University, Wenzhou, 325000 China

**Keywords:** FGF1, Acute lung injury, Lipopolysaccharide, Inflammation, Oxidative stress, TLR4, Nrf2, NF-κB

## Abstract

**Background:**

Acute lung injury (ALI) and its severe form, acute respiratory distress syndrome (ARDS), are devastating clinical disorders with high mortality, and for which more effective therapies are urgently needed. FGF1, the prototype member of the FGF family, is shown to exert protective effects against injurious stimuli in multiple disease models. Here we aimed to evaluate whether FGF1 pretreatment is protective against LPS-induced ALI and elucidate the potential underlying mechanisms.

**Methods:**

For drug-treated groups, C57B/6 mice received a single i.p. injection of FGF1 (1 mg/kg) 1 h before the LPS challenge or not. To induce the ALI model, the mice were treated by intratracheal instillation of LPS (5 mg/kg). Then, histopathological changes in lung tissues were assessed by hematoxylin and eosin staining and transmission electron microscopy. ELISA and qPCR assays were used to detect pro-inflammatory cytokine levels in BALF and lung tissues, respectively. The total number of inflammatory cells (neutrophils and macrophages) in BALF were counted using the Wright-Giemsa method. The expressions of reactive oxygen species (ROS) and malondialdehyde (MDA) were measured using their respective kits. Western blot and immunostaining were used to evaluate the expressions of antioxidants (Nrf-2, HO-1, SOD2, GPX4, and Catalase), as well as the inflammatory and/or apoptosis-related factors (TLR4, NF-κB, and Cleaved- caspase 3).

**Results:**

FGF1 pretreatment significantly ameliorated the LPS-induced histopathological changes, reduced lung wet/dry ratios, ROS and MDA levels, total BALF protein, inflammatory cell infiltration, proinflammatory cytokine levels, and significantly increased the expression of antioxidant proteins (Nrf-2, HO-1, Catalase, and SOD2). In addition, FGF1 pretreatment significantly reduced the expression of TLR4 and cleaved- caspase 3, inhibited NF-κB activation, and reduced LPS-induced cell apoptosis.

**Conclusions:**

Altogether, our results suggest that FGF1 pretreatment is protective against LPS-induced ALI through mediating anti-inflammatory and antioxidant effects, which may be attributed to the downregulation of TLR4 expression and inhibition of NF-κB activation, as well as promotion of antioxidant defenses. Therefore, FGF1 administration may prove beneficial in preventative strategies for ALI/ARDS.

**Supplementary Information:**

The online version contains supplementary material available at 10.1186/s10020-022-00502-8.

## Background

Acute lung injury (ALI) and its more severe form, acute respiratory distress syndrome (ARDS), are devastating clinical disorders characterized by widespread inflammation, diffuse alveolar damage, pulmonary edema, often leading to respiratory failure and death (Matthay et al. 2012; Saguil and Fargo 2012). ALI pathogenesis involves multiple mechanisms, including oxidative stress and inflammation. Oxidative stress, manifested by aberrant production of oxidants (ROS) and/or impaired antioxidant defense(s), can cause cell injury, triggering inflammatory cell-mediated accumulation of proinflammatory cytokines such as TNF-α, IL-6, and IL-1β (Bloomer 2008; Tomashefski Jr 2000).

LPS, an endotoxin of gram-negative bacteria, can induce ALI via TLR4-mediated oxidative stress and inflammation (Chow et al. 1999) and is commonly used to induce ALI in animal models. Furthermore, LPS elicits infiltration of inflammatory cells, such as neutrophils and macrophages, into the injured lungs, further aggravating inflammatory response. To date, therapies for managing ALI lack efficacy and can cause serious side effects. Therefore, an urgent need remains for the development of novel therapeutic strategies with minimal side effects.

FGF1, the prototype member of the FGF family, signals through binding all FGFRs (FGFR1-FGFR4) (Zhang et al. 2006), and serves as a potent mitogen for both mesenchymal and epithelial cell compartments of the lung (Lebeche et al. 1999). Besides lung-resident cell localization, FGFRs are also expressed in various other cell types, including infiltrating neutrophils and macrophages, endowing them with the capacity to directly respond to FGF1 signaling. For instance, neutrophils were shown to predominantly express FGFR2 on their cell surface, whereas FGFR1 and 4 localized in the cytosol (Haddad et al. 2011; Wang et al. 2018). Furthermore, FGFR1-4 expression was detected in both mouse lung and RAW264.7 macrophages, further implicating FGF signaling in the regulation of these cells’ activities (Wang et al. 2019).

FGF1-FGFR signaling has been implicated in diverse physiological and pathological processes ranging from lung morphogenesis (Morrisey and Hogan 2010), bone formation (Dunstan et al. 1999), metabolic regulation (Suh et al. 2014), wound healing (Sahni et al. 1999) to tumor biology (Ding et al. 2003). Importantly, FGF1 was shown to exert protective effects against oxidative stress and inflammation in various disease models, including spinal cord injury (Vargas et al. 2005) and diabetic nephropathy (Liang et al. 2018), via activation of Nrf2- mediated antioxidant defense and suppression of NF-κB activation, respectively. Given these findings, whether FGF1 signaling may also prove beneficial in the context of ALI/ARDS remains unexplored. Hence, in this study, we evaluated whether FGF1 pretreatment is protective in ALI by attenuating LPS-induced oxidative stress and inflammation.

## Materials and methods

### Reagents and antibodies

LPS (Escherichia coli 0111: B4) was purchased from Sigma (St. Louis, MO, USA). FGF1 (purity > 99%) was purchased from Zhejiang Grost Biotechnology (Wenzhou, China). The following antibodies were used to detect the proteins of interest: TNF-α (Abcam; Cat# ab1793), phospho-FGFR1 (Abcam; Cat# ab173305), Nrf2 (Cell Signaling Technology; Cat# 12,721 S), NF-κB p65 (Cell Signaling Technology; Cat# 8242 S), HMGB1 (Cell Signaling Technology; Cat# 6398 S), IL-1β (Bioworld Technology; Cat# BS6067), IL-6 (Cell Signaling Technology; Cat# 12912T), Laminin B (Santa Cruz Biotechnology; Cat# sc-377,000), β-actin (Santa Cruz Biotechnology; Cat# sc-47,778), SOD2 (Santa Cruz Biotechnology; Cat# sc-137,254), HO-1 (Santa Cruz Biotechnology; Cat# sc-136,960), Catalase (Abcam; Cat# ab52477), GPX4 (Abcam; Cat# ab12506), TLR4 (Abcam; Cat# ab13556), cleaved-caspase 3 (Abcam; Cat# ab2302), phospho-IκB (Santa Cruz Biotechnology; Cat# sc-8404), IκB (Santa Cruz Biotechnology; Cat# sc-1643), Goat Anti-Rabbit IgG (H + L) HRP (Bioworld, BS13278), Goat Anti-Mouse IgG (H + L) HRP (Bioworld, BS12478), Donkey anti-Mouse IgG Alexa Fluor^®^ 568 (Abcam, Cat# ab175707), Donkey anti-Mouse IgG Alexa Fluor^®^ 488 (abcam, Cat# ab150077). Mouse TNF-α, IL-1β, and IL-6 ELISA kits were purchased from Invitrogen (Vienna, Austria). Malondialdehyde (MDA) determination kit was provided by the Beyotime (Shanghai, China). RIPA buffer (CAS: R0020), Phosphatase inhibitors (CAS: P1260), Triton X-100 (CAS: 9002-93-1), 4% Paraformaldehyde (CAS: P1110), and DAPI (CAS: 28718-90-3) were purchased from Solarbio LIFE SCIENCES (Beijing, China). The terminal deoxynucleotidyl mediated dUTP nick-end (TUNEL) Assay kit was purchased from Vazyme biotech^®^ China.

### Animals

Eight-week-old male mice C57BL/6 weighting 20–25 g (Animal Center of the Chinese Academy of Sciences in Shanghai, China) were used in the study. The animals were housed in a temperature and humidity-controlled environment with a 12 h light/12 h dark cycle. All the experimental procedures were performed in compliance with the National Institutes of Health guidelines and with approval from the Animal Care and Use Committee of Wenzhou Medical University, China (Ethics number: wydw2019-0510).

### Murine model and experimental design

**Fig. 1:**
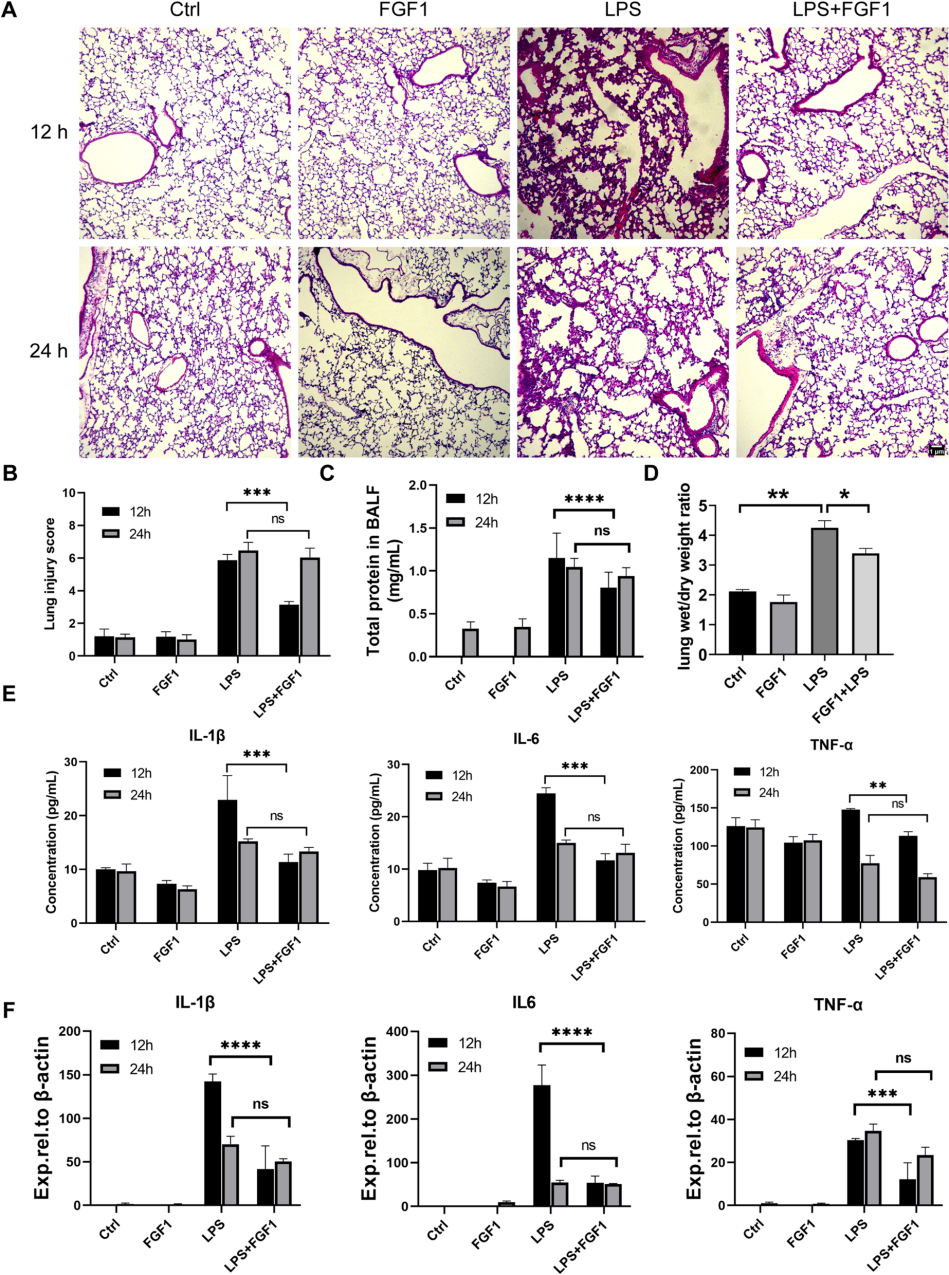
FGF1 pretreatment protects against LPS-induced ALI. **A** H&E staining of lung sections at 12 and 24 h after LPS stimulation, magnification (10 ×). **B** Histological mean lung injury scores from 28 lung sections (n = 4 per group). **C**, **D** Total BAL protein and lung wet/dry weight ratios were evaluated at 12 h post LPS treatment. **E**, **F** The mRNA expression of TNF- α, IL-6 and IL-1β were determined at 12- and 24 h time points. Data are presented as mean ± SD (n = 4 per group). *p < 0.05, **p < 0.01, ***p < 0.001, ****p < 0.0001; One-way or Two-way ANOVA with Tukey post hoc-analysis were applied where appropriate

Mice were randomly divided into four groups with 4–5 animals per group. Mice were anesthetized with an i.p. injection of 1% pentobarbital sodium (50 mg/kg, Merck, Germany). After that, LPS was intratracheally delivered into the lungs of mice at doses of 5.0 mg/kg body weight to induce the ALI model. As for FGF1-treated groups, FGF1 solution was i.p. injected at 1.0 mg/kg for 1 h before LPS administration or not. After LPS treatment for 12 and 24 h (Figs. 1, 5), the mice were euthanized by diethyl ether, then the lung tissues were collected from different groups. For the collection of bronchial alveolar lavage fluid (BALF), we injected 0.5 ml PBS into the trachea and aspirate the liquid three times to obtain BALF (Kuo et al. 2011). The BALF liquid was centrifuged at 3000 rpm for 10 min at 4 °C, then the supernatants were used for detection of cytokines, and the sediment cells were used for inflammatory cell counting by Wright–Giemsa staining. The total protein in BALF was quantified using the Solarbio® BCA kit (Cat# PC0020), as per the manufacturer’s instructions.

**Fig. 2:**
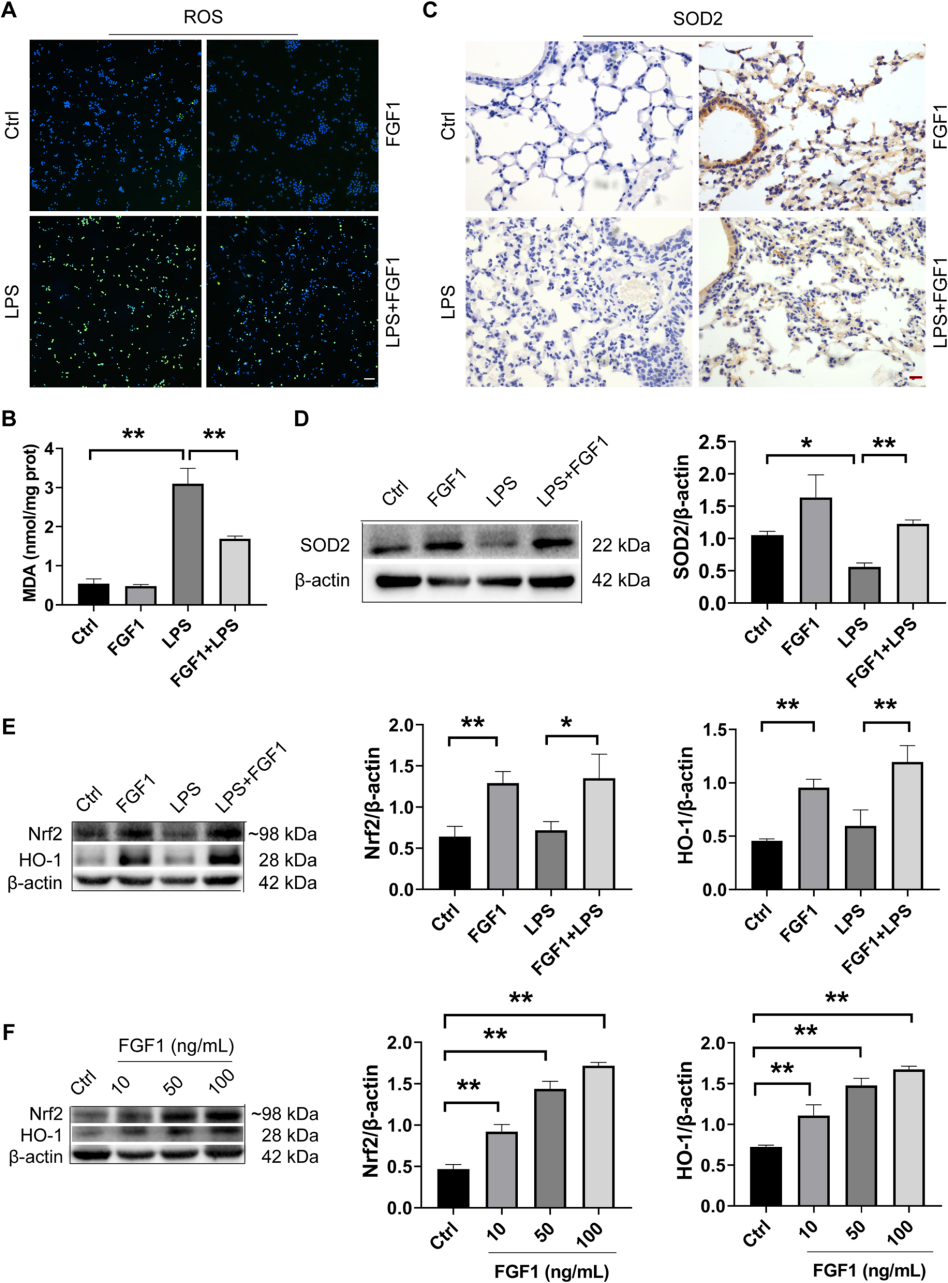
FGF1 pretreatment ameliorates LPS-induced oxidative stress via promoting antioxidant defenses. Effects of FGF1 pretreatment on ROS (**A**), MDA (**B**), in RAW264.7 cells and mice lung tissues, respectively; scale bar = 64 μm. **C**, **D** Immunohistochemistry and WB analysis of SOD2 in lung tissues; scale bar = 64 μm. **E**, **F** WB analysis of Nrf2, HO-1 in mice, and cells incubated with varying concentrations of FGF1, respectively. All data were analyzed at 12 h after LPS treatment. Data are presented as mean ± SD (n = 5 per group). *p < 0.05, **p < 0.01; One-way ANOVA with Tukey’s post-hoc test

### Cell culture and treatment

RAW264.7 and A549 cells were obtained from the China Cell Line Bank (Beijing, China). The cells were cultured in medium (DMEM) supplemented with 10% fetal bovine serum (FBS) in a humidified incubator containing 5% CO_2_ at 37 °C. Cells were grown in plates and allowed to acclimate for 24 h before further treatment. For WB analysis, RT-qPCR, and immunofluorescence assay, the cells were placed in RPMI with reduced FBS (0.5%) and pretreated with FGF1 (100 ng/mL) for 1 h. After that, cells were exposed to LPS (1 µg/mL) for 12 h.

### Histological analysis and immunohistochemistry

For microscopic examination, the mice were euthanized at 12 and 24 h after the LPS challenge, and the lungs of mice were excised. The lungs were next fixed in 4% paraformaldehyde solution overnight, then gradually dehydrated in ethanol, and processed for paraffin embedding in paraffin and cut into 5-µm-thick sections. After deparaffinization and rehydration, the tissues were stained with hematoxylin and eosin (H&E), and we used a five-point scale from 0 to 4 to determine the lung injury score.

For transmission electron microscopy, the slices were first fixed with 2.5% glutaraldehyde in phosphate buffer (PH = 7.0) for 4 h, then post-fixed with 1% osmium tetroxide (PH = 7.0) for l h and washed three times in the phosphate buffer. The slices were embedded in epoxy resin after dehydration. Ultrathin sections were counterstained with uranyl acetate and alkaline lead citrate for 15 min, respectively and observed in Transmission electron microscopy (TEM, Hitachi TEM system, Tokyo, Japan).

The expression of phospho-FGFR1, TNF-α, IL-1β (data not shown), and SOD2 in the lung tissues of each group was conducted with immunohistochemistry. The slides were incubated with primary antibodies at 4 °C for 12 h. After washing, the secondary antibody was added and incubated for 1 h. Subsequently, the sections were stained with Diaminobenzidine (DAB) and counterstained with hematoxylin.

### Lung wet/dry (W/D) weight ratio

The lung tissues were excised 12 h after the LPS challenge and the wet weight was recorded immediately. For dry weight determination, the lungs were incubated at 60 °C for 24 h. Subsequently, the ratios of wet-to-dry weight were calculated for the assessment of tissue edema.

### Gene-expression analysis

Total RNA was extracted from lung tissues using TRIzol (Invitrogen), and reverse transcribed into cDNA by employing the Prime-Script RT-PCR kit (Takara) following the manufacturer’s instructions. Subsequently, quantitative PCR (qPCR) was performed using the SYBR Green Quantitative PCR kit (Bio-Rad) reagents for expression of TNF-α, IL-1β, and IL-6 with primers as previously reported (Tan et al. 2017). The target values were normalized to β-actin (forward: 5′-AGAGGGAAATCGTGCGTGAC-3′, reverse: 5′-CAATAGTGATGACCTGGCCGT-3′).

### Western blot analysis

The protein extraction was performed from lung tissues or cultured cells for western blot (WB) analysis. All the samples were lysed in RIPA buffer with protease and phosphatase inhibitors for 20 min and centrifuged. The supernatants were collected. Subsequently, protein concentrations were measured with a Bradford protein assay kit. Proteins were separated by 10% SDS-PAGE and then transferred to a PVDF membrane for WB with primary antibodies against TNF-α, Nrf2, HMGB1, NF-κB p65, IL-1β, IL-6, IκB-α, β-actin, SOD2, HO-1, phospho-IκB, Catalase, GPX4, TLR4, cleaved caspase-3 and Laminin B. Then the membranes were incubated with corresponding HRP-conjugated secondary antibodies for 1 h. After that, the bands were detected with the enhanced chemiluminescence (ECL) detection system (BI, EZ-ECL) according to the manufacturer’s instructions, and the bands were visualized by ChemiDoc TM XRS + imaging system (Bio-Rad Laboratories, USA). The protein densities were quantified by Image Lab (Bio-Rad, version 5.2) and ImageJ.

### Immunofluorescence assay

To assess the effect of FGF1 on the suppression of TNF-α in vitro, A549 cells (10^5^ cells/well) were grown in glass-bottom dishes. After FGF1 and LPS administration, the cells were fixed and treated for 5 min with 0.3% Triton X-100. Afterward, the cells were blocked by BSA (5%) for 1 h. Subsequently, the cells were incubated with antibodies against TNF-α and NF-κB p65 overnight at 4 °C. The cells were then incubated with the secondary antibodies (Alexa Fluor^®^ 568 or Alexa Fluor^®^ 488) for 1 h at room temperature. DAPI was used for nuclear counterstaining. The cells were observed and photographed using a Nikon Eclipse Ti-U inverted fluorescence microscope. For DHE staining, frozen section (5 μm) of lung tissues were incubated with DHE (Beyotime; Cat# S0063) for 30 min and visualized using a fluorescence microscope (Nikon). For the quantification of nuclear NF-κB p65 in A549 cells, the confocal microscopy data were quantified by ImageJ software, and the corrected total cell fluorescence (CTCF) was calculated according to the formula: CTCF = integrated density (area of selected region × mean fluorescence of background readings).

### TUNEL assay

Lung sections were prepared as described earlier, and cell apoptosis was detected by TUNEL using the Vazyme^®^ TUNEL BrightRed Apoptosis Detection kit (A113). TUNEL-positive cells were counted following the manufacturer’s instructions.

### Cytokine quantification via enzyme-linked immunosorbent assay (ELISA)

After LPS was administered for 12 and 24 h, BALF was collected by intra-tracheal instillation of 0.5 ml PBS and gentle aspiration 3 times (Kuo et al. 2011). The BALF liquid was centrifuged at 3000 rpm for 10 min at 4 °C, then the supernatants were used for detection of cytokines, and the sediment cells were used for inflammatory cell counting by Wright–Giemsa staining. The total protein in BALF was quantified using the Solarbio® BCA kit (Cat# PC0020), as per the manufacturer’s instructions.

### Determination of MDA contents

The lungs and cells were homogenized and levels of MDA were measured using test kits according to the manufacturer’s instructions (Shanghai Beyotime Biotechnology).

### Measurement of ROS production

For intracellular ROS determination, RAW264.7 cells were seeded into 96-well plates (10^4^ cells/well) for 24 h incubation, then transferred into serum-free DMEM with FGF1 (100 ng/mL) for 1 h and stained with DCFH-DA (50 mM) for 30 min. After that, the cells were washed with PBS twice and t-BHP (10 mM) was used to induce cells generating ROS, which were measured by ROS Assay Kit (Beyotime Biotechnology, S0033S) according to the manufacturer’s recommendations.

### Statistical analysis

All data are shown as means ± SEM. The results were analyzed using GraphPad Prism software (Graph Pad Software Inc. USA). Analysis of variance (ANOVA) tests were used for comparison among groups. *p < 0.05, **p < 0.01, ***p < 0.001, or ****p < 0.0001 indicates the level of statistical significance.

## Results

### FGF1 pretreatment protects against LPS-induced ALI

To investigate the potential effects of FGF1 on LPS-induced ALI, mice received a single i.p. injection of FGF1 1 h prior LPS instillation. Then, lung tissues and BALF were collected to evaluate lung injury severity, proinflammatory cytokine- and total protein levels at 12- and 24 h time-points (Additional file 1: Fig. S1A). LPS-exposed mice, when compared to vehicle and FGF1 groups, exhibited significant pathological changes such as alveolar hemorrhage, edema, thickened alveolar walls (Fig. 1A, B; Additional file 1: Fig. S1B), elevated BALF protein levels (Fig. 1C), and increased lung wet/dry ratio (Fig. 1D). However, FGF1 pretreatment significantly improved these pathological changes. Consistent with H&E and TEM (Additional file 1: Fig. S1B) analyses, qPCR and ELISA analysis revealed LPS-induced significant increase in expressions of proinflammatory cytokines TNF-α, IL-6, and IL-β, which were attenuated by FGF1 pretreatment (Fig. 1E, F). Notably, FGF1 administration exerted its most potent protective effects on LPS-induced ALI at 12 h, as these effects could not be sustained at 24 h. Overall, these results suggest that FGF1 ameliorated LPS-induced ALI, as evidenced by improvement of pathologic changes and reductions in proinflammatory cytokine levels in murine lungs, and total BALF protein levels; thus, suggesting therapeutic potential for improving ALI.

### FGF1 pretreatment counteracts LPS-induced oxidative stress via enhancing Nrf-2-mediated antioxidant defenses

Increased oxidative stress, characterized by an imbalance between oxidants (ROS and MDA) and antioxidants (HO-1, SOD2, CAT) in favor of the oxidants, is a major contributing factor to the pathogenesis of ALI. Here we evaluated the effect of FGF1 on LPS-induced oxidative stress and the expression of the antioxidant proteins in vivo and in vitro. As shown in (Fig. 2A, B), FGF1 pretreatment could significantly reduce ROS and MDA production in lung tissues from LPS-challenged mice, as well as in RAW264.7 cells (data not shown). By contrast, pretreatment with FGF1 significantly increased the expression of antioxidants tested (HO-1, SOD2) (Fig. 2C, D–F), Catalase (Additional file 2: Fig. S2) except for GPX4, whose expression showed a statistically nonsignificant increase, compared to the LPS-treated group (Additional file 2: Fig. S2). Additionally, we sought to test whether FGF1 could influence the expression of Nrf-2, an important upstream regulator of cellular redox balance and antioxidant transcription (HO-1, SOD2, GPX4, and CAT). As shown in (Fig. 2E, F), FGF1 pretreatment significantly upregulated Nrf2 expression in the LPS + FGF1 group, compared to the control and LPS-alone group. Taken together, these results suggest that FGF1 may counteract LPS-induced oxidative stress through the enhancement of endogenous antioxidant defenses.

**Fig. 3:**
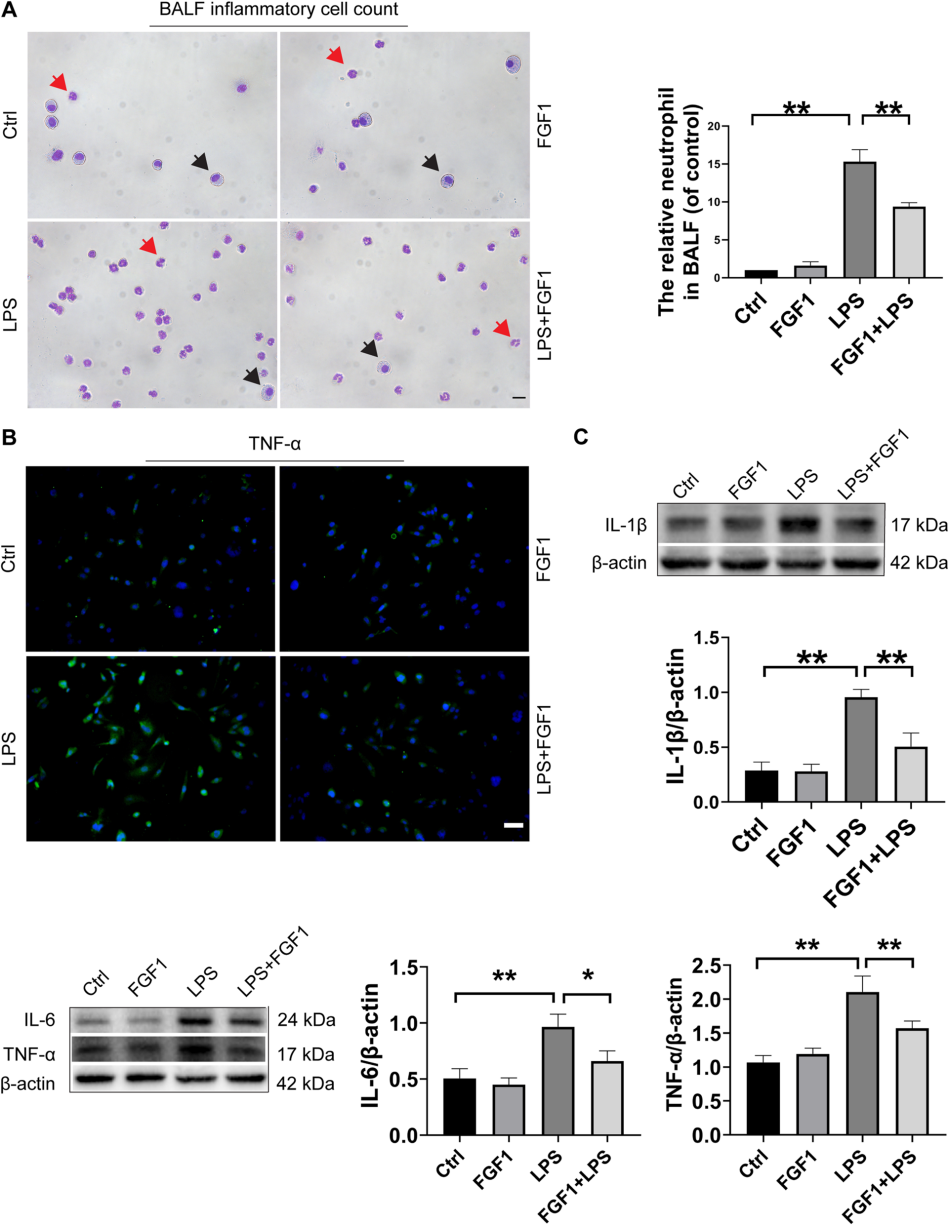
FGF1 pretreatment dampens LPS-induced inflammatory cell infiltration and cytokine production. **A** Inflammatory cell infiltration was quantified by counting the total number of inflammatory cells, neutrophils, and macrophages in BALF using the Wright-Giemsa method (red arrows indicate the neutrophils and black arrows indicate the macrophages). Scale bar: 64 μm, magnification: 40×. **B** Representative immunofluorescence analysis of A549 cells for TNF-α expression, (scale bar = 64 μm, magnification: 10×). **C** WB analysis and quantification of the relative protein expression of murine IL-6, IL-1β, and TNF-α; β-actin was used for normalization. Data are presented as mean ± SD (n = 3 to 5 per group). *p < 0.05, **p < 0.01; One-way ANOVA with Tukey’s post-hoc test

### FGF1 pretreatment dampens LPS-induced inflammatory cell infiltration and proinflammatory cytokine release

Excessive inflammatory cell infiltration and aberrant secretion of cytokines are closely linked with ALI pathogenesis (Abraham et al. 2006; Rittirsch et al. 2008). Here we investigated the potential effects of FGF1 on LPS-induced inflammatory cell infiltration into the lung tissue and proinflammatory cytokine release at 12 h. BALF analysis revealed that lung tissue-infiltrating inflammatory cells were especially neutrophils, and their numbers decreased upon pretreatment with FGF1 (Fig. 3A). Furthermore, FGF1 significantly reduced the inflammatory cell-derived proinflammatory cytokines TNF-α, IL-6, and IL-1β in mice (Fig. 3C). Consistent with these findings, qPCR analysis of a macrophage cell line RAW264.7 showed significantly increased mRNA expression of TNF-α, IL-6, and IL-1β in LPS-treated cells, whereas FGF1 pretreatment counteracted the increases (data not shown). Additionally, immunofluorescence staining of TNF-α in A549 cells similarly revealed the protective effects of FGF1 against inflammatory damage (Fig. 3B). Overall, these results suggest that FGF1 may dampen LPS-induced infiltration of inflammatory cells, as well as associated production of proinflammatory cytokines in ALI.

**Fig. 4:**
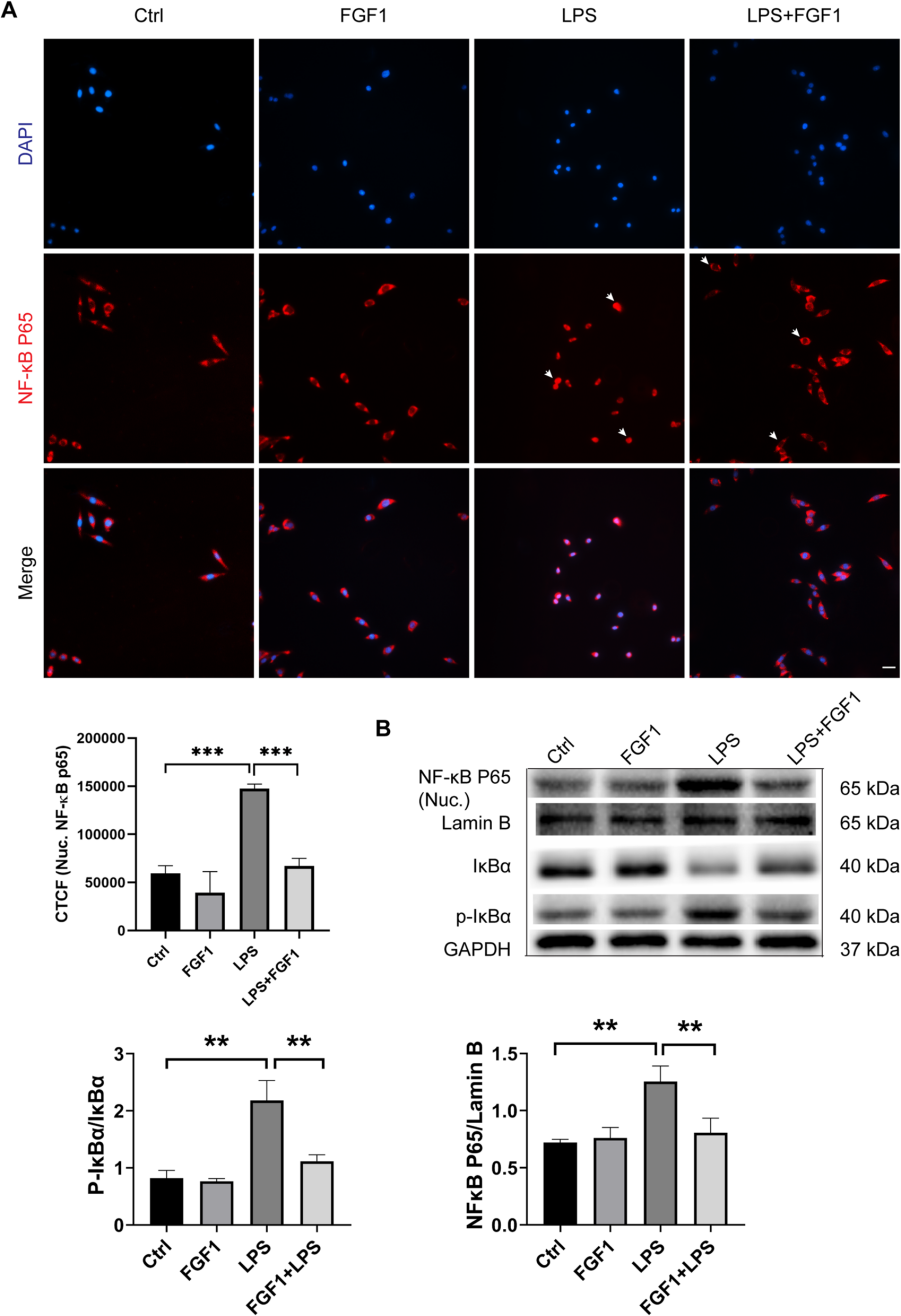
FGF1 pretreatment inhibits NF-κB nuclear translocation. **A** Immunofluorescence analysis and quantification of NF-κB p65 nuclear expression in A459 cells across different groups (scale bar = 64 μm). The fluorescence levels were quantified using ImageJ software. Corrected Total cellular Fluorescence (CTCF) = integrated density (area of selected region × mean fluorescence of background readings). **B** The relative protein expression levels of nuclear NF-κB p65, Lamin B, IκBα, p-IκBα were analyzed by western blot. Bar graphs show the quantification of protein expression levels normalized to GAPDH. All data were analyzed at 12 h after LPS treatment. Data are presented as mean ± SD (n = 3 to 5 per group). **p < 0.01, ***p < 0.001; One-way ANOVA with Tukey’s post-hoc test

### FGF1 pretreatment suppresses nuclear translocation and activation of NF-kB in LPS-induced ALI

NF-κB activation, which occurs primarily via IKK-dependent release of the IκBα-bound NF-κB p65 subunit for subsequent nuclear translocation, triggers transcription of most pro-inflammatory genes including TNF-α, IL-6, and IL-1β. In this regard, aberrant activation of NF-κB is considered a hallmark event in the pathogenesis of ALI. To further elucidate the potential mechanisms underlying the anti-inflammatory effects of FGF1 (Fig. 3A), we examined whether FGF1 pretreatment could suppress NF-κB activation in LPS-induced ALI at 12 h. As shown in (Fig. 4B), LPS-treatment significantly increased IκBα phosphorylation, indicating dissociation of the IκBα/NF-κB p65 complex and subsequent nuclear translocation of NF-kB p65 in vivo. By contrast, FGF1 pretreatment significantly reduced the LPS-induced increase in p-IκBα levels. Consistent with the p-IκBα WB results, Immunofluorescence (A549 cells) and WB (mice lung tissues) assays revealed that LPS significantly enhanced nuclear translocation of NF-κB p65, whereas pretreatment with FGF1 reduced the nuclear p65 levels (Fig. 4A). Altogether, these results suggest that the anti-inflammatory effects of FGF1 in LPS-induced ALI may be mediated via inhibition of the NF-κB nuclear translocation, as well as subsequent activation of proinflammatory genes.

**Fig. 5:**
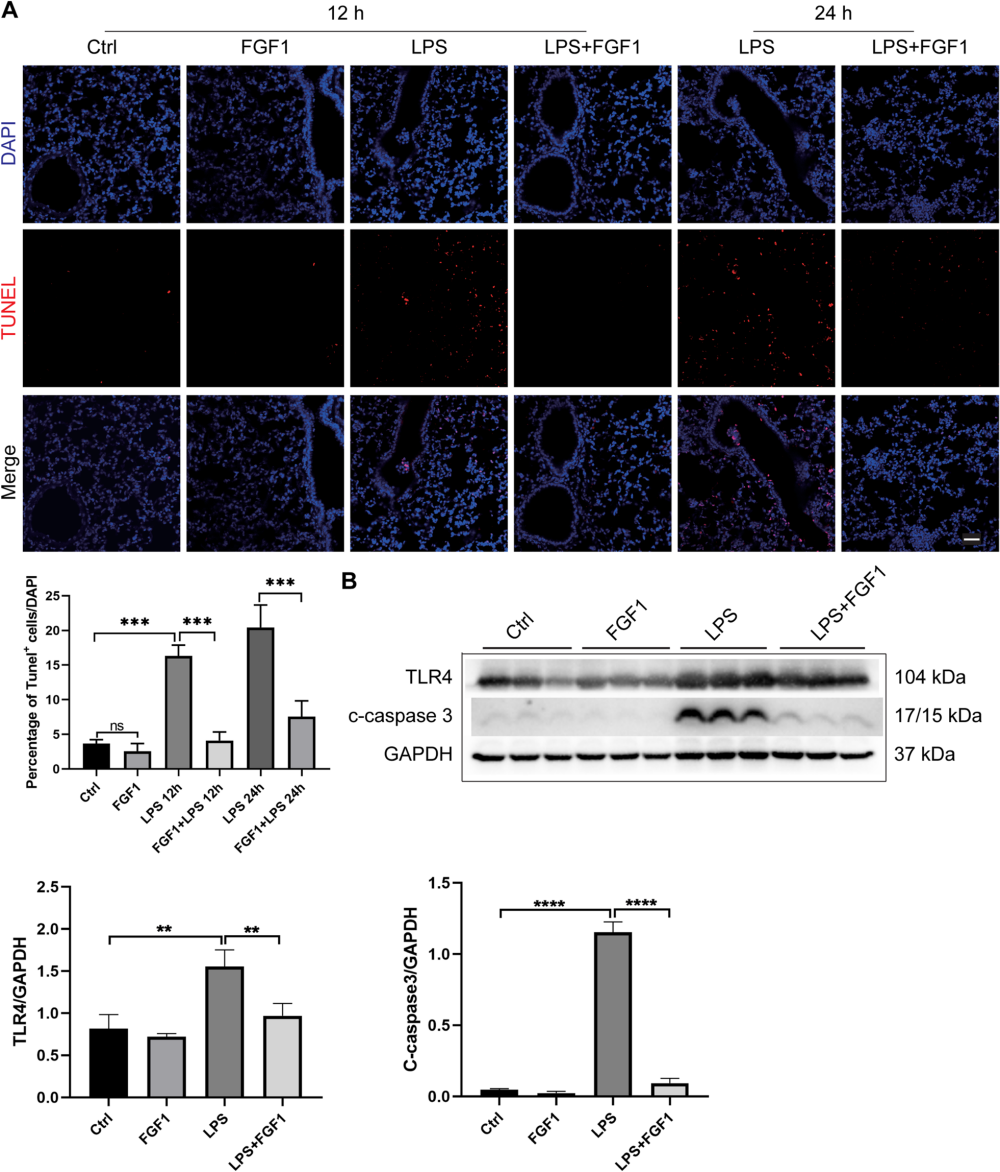
The effects of FGF1 pretreatment of LPS-induced cell death. **A** Representative images of TUNEL assay staining to detect apoptotic cells in the lung sections of mice at 12- and 24 h. Scale bar = 100 μm. Apoptotic cells were observed under fluorescence microscopy and the percentage of TUNEL positive cells per 40x magnification field was calculated. **B** WB analysis and quantification of TLR4 and the downstream apoptosis-related protein cleaved-caspase 3 in murine lungs. WB assays were performed at 12 h post LPS treatment. Data are presented as mean ± SD (n = 3 per group). **p < 0.01, ***p < 0.001, ****p < 0.0001; One-way ANOVA with Tukey’s post-hoc test

### The effects of FGF1 pretreatment on LPS-induced cell death in ALI

LPS is known to exert its effects via TLR4, one of the most potent inducers of NF-κB- mediated inflammation and cell apoptosis (Gao et al. 2020; Jung et al. 2005; Kawai and Akira 2007). To investigate whether FGF1 pretreatment has protective effects against LPS-induced apoptosis in ALI, we examined the protein expressions of TLR4 and caspase 3, the main executioner protease of apoptotic cell death acting downstream of TLR4 signaling, at 12 h. Additionally, apoptotic cells in ALI mice were detected using the TUNEL assay. As shown in (Fig. 5B), we found that LPS significantly increased TLR4 and cleaved caspase 3 protein levels in the LPS group compared to the control group, whereas FGF1 pretreatment effectively reduced the LPS-induced increases in protein expression. Consistently, the TUNEL assay also revealed increased apoptosis in response to LPS-induced injury, while FGF1 pretreatment significantly reduced the number of TUNEL positive cells (Fig. 5A). Overall, these results together with the above findings suggest that FGF1 exerts protective effects against LPS-induced cell death, likely via downregulation of the TLR4/NF-κB/c-caspase 3 pathway.

## Discussion

This study revealed the cytoprotective effects of FGF1 in LPS-induced ALI mice, RAW264.7, and A549 cells, which may be attributed to the downregulation of LPS/TLR4-mediated oxidative stress and inflammation. Mechanistically, FGF1 pretreatment significantly inhibited TLR4/NF-κB-mediated pro-inflammatory cytokine release and cell apoptosis and promoted Nrf2-driven antioxidant defenses. Moreover, FGF1 pretreatment significantly mitigated LPS-induced histopathological changes in murine lung tissues. To our knowledge, these findings provide the first evidence that FGF1 pretreatment is protective against LPS-induced ALI, suggesting that FGF1 administration may prove beneficial in preventative strategies for ALI/ARDS.

In LPS-induced ALI, TLR4-mediated oxidative stress and inflammation perturb alveolar-capillary barrier integrity, thereby increasing barrier permeability with the subsequent influx of inflammatory cells and protein-rich edema fluid into the alveolar space (Johnson and Matthay 2010; Rittirsch et al. 2008). This accounts for the accumulation of proinflammatory cytokines in BALF. In this study, although ELISA and q-PCR analysis showed an overall reduction in the proinflammatory cytokine levels in lungs/ BALF with FGF1 pretreatment at both 12 and 24 h time points, the reduction was only statistically significant at 12 h (Fig. 1E, F). Consistently, histopathological analysis (Fig. 1A–D) also showed that the protective effects of FGF1 pretreatment against LPS-induced ALI were not sustainable at 24 h. Plausibly, the observed transient protective effects of FGF1 may be attributed to the use of a single FGF1 dose, given its short biological half-life.

TLR4-mediated generation of oxidants and free radicals, especially ROS, (Ikram et al. 2019; Park et al. 2004; Sasaki et al. 2008; Zhang et al. 2021) represents a key event leading to oxidative stress and subsequent cell injury/apoptosis. Excessive ROS levels beget oxidative stress via several mechanisms, including inactivation of antioxidant defenses (Nrf2and Nrf-2- dependent factors HO-1, SOD2, and Catalase) (Bhattacharya 2015). By contrast, effective antioxidants prevent ROS-mediated oxidative damage by scavenging the free radicals/ROS. In the present study, FGF1 pretreatment significantly enhanced the expressions of endogenous antioxidants (Nrf-2, HO-1, Catalase, and SOD2), except for GPX4, whose expression showed a statistically nonsignificant increase, compared to the LPS-treated group.

Aberrant NF-κB activation contributes to the development of ALI, among other inflammatory disorders, by mediating the transcription of proinflammatory cytokines such as TNF-α, IL-1β, and IL-6, which in turn enhance the inflammatory response (Do-Umehara et al. 2013). Here FGF1 pretreatment significantly suppressed LPS/TLR4-induced production of TNF-α, IL-6, and IL-1β in mice, via inhibition of NF-κB activation, which is consistent with previous report (Liang et al. 2018). Similar results were obtained in cultured RAW 264.7, a widely used mouse macrophage cell line, and human epithelial cell line A549, further validating the role of FGF1 in mediating anti-inflammatory effects by suppressing NF-κB activity. Taken together, our findings are in general consistency with an earlier study by Vargas showing FGF1-mediated activation of antioxidants Nrf2 and HO-1 in astrocytes (Vargas et al. 2005), and two recent studies demonstrating the protective effect of FGF1 against diabetic nephropathies (Liang et al. 2018), and a mutant FGF1 with reduced mitogenic activity against the development of chronic kidney disease induced by either diabetic- or adriamycin-induced nephropathy via activation of downstream AMPK/GSK-3β/Nrf2 and suppression of the JNK/NF-κB, respectively (Wang et al. 2019).

Although we believe that our current results, together with previous studies (Liang et al. 2018; Vargas et al. 2005), sufficiently support the conclusions drawn regarding the protective effects of FGF1 against oxidative stress and inflammation, our study suffers from a number limitations, one of which is the lack of genetic knockout models. Thus, further studies using particularly Nrf-2/NF-κB knock-out (KO) models are warranted to further validate and expand upon the current findings. Furthermore, despite recapitulating several key pathological features of human ALI/ARDS tested by our hypothesis, the LPS-induced ALI model is associated with several caveats. For instance, although LPS administration does induce an inflammatory response with neutrophil accumulation in mice lungs, changes in alveolar-capillary permeability are subtle relative to patients (Matute-Bello et al. 2008). In addition, neutrophils represent approximately 50–70% of all circulating leukocytes in humans, which is much higher when compared to mice (10–25%) (Mestas and Hughes 2004). Thus, the large interspecies difference in the circulating neutrophil count, as well as the mild alveolar-capillary permeability changes reflect the inability of this model to fully reproduce certain features of ALI/ARDS observed in humans, including severe neutrophilic alveolitis. Moreover, the relatively small size of rodents, among other fundamental disparities, places caveats on sample sizes that can be collected/harvested from mice, such as BALF, and hence serves as a potential source of imprecision in experimental data analysis and interpretation. Altogether, these limitations may, in part, explain why many pharmacological interventions that showed promises in preclinical ALI/ARDS failed to translate into novel therapies. Therefore, robust preclinical models that recapitulate human ALI/ARDS at an even higher degree are required to improve the translatability of preclinical findings into the clinic.

While many preclinical studies, including our present one, have shown FGF1 to be efficacious in various pathological models of human diseases, some major hurdles remain before these findings can be translated into the clinic as FGF1 is associated with several adverse effects. For instance, increased FGF1 expression levels were observed in IPF lung regions undergoing active remodeling, suggesting that aberrant FGF1 signaling may facilitate progressive lung remodeling in IPF (MacKenzie et al. 2015). In addition, recent studies revealed that FGF1 may promote the amplification and stemness properties of lung and pancreatic cancer cells (Liu et al. 2017; Quan et al. 2020).Thus, to optimize the applicability and efficiency of FGF1, future studies should aim to, at least minimize these adverse effects.

In summary, the present study suggests that FGF1 is protective against LPS/TLR4-mediated oxidative damage and inflammatory response via upregulation of antioxidant defenses and suppression of NF-κB activation.

## Conclusions

Our findings not only provide the first evidence that FGF1 alleviates LPS-induced ALI in murine lungs, but also raise the potential that FGF1-based therapies may be an attractive preventative strategy for ALI/ARDS.

## Supplementary information

**Additional file 1**: **Figure S1.** (A) Schematic of the experimental procedure with a timeline. (B) Representative transmission electron micrographs showing ultrastructural changes in LPS-induced ALI, taken at 12 h. The scale bar is 5 μm. Blue arrows indicated alveolar-capillary barrier, yellow arrows indicated edema and thickened septa containing infiltrates, red asterisk indicated disrupted endothelial-capillary barrier. Mean (SEM), n = 5 per group. ATII denoted alveolar type II cells; lb, lamella bodies; RBC, red blood cell; N, neutrophil; A.s, alveolar space, respectively. 

**Additional file 2**: **Figure S2.** (A) Western blot analysis and quantification of the relative protein expressions of antioxidant enzymes Catalase and GPX4 normalized to GAPDH. Data are presented as mean ± SD (n = 5 per group). *p < 0.05; One-way ANOVA with Tukey’s post-hoc test. 

## Data Availability

Not applicable.
