# Supplementary material for: FGF1 alleviates LPS-induced acute lung injury via suppression of inflammation and oxidative stress
Source: Mol Med. 2022 Jun 28;28:73. doi: 10.1186/s10020-022-00502-8 (PMC9238076; doi:10.1186/s10020-022-00502-8)
Supplement: Supplementary file 1 — Additional file 1: Figure S1. (A) Schematic of the experimental procedure with a timeline. (B) Representative transmission electron micrographs showing ultrastructural changes in LPS-induced ALI, taken at 12 h. The scale bar is 5 μm. Blue arrows indicated alveolar-capillary barrier, yellow arrows indicated edema and thickened septa containing infiltrates, red asterisk indicated disrupted endothelial-capillary barrier. Mean (SEM), n = 5 per group. ATII denoted alveolar type II cells; lb, lamella bodies; RBC, red blood cell; N, neutrophil; A.s, alveolar space, respectively. [file 10020_2022_502_MOESM1_ESM.docx]

­­­­­­


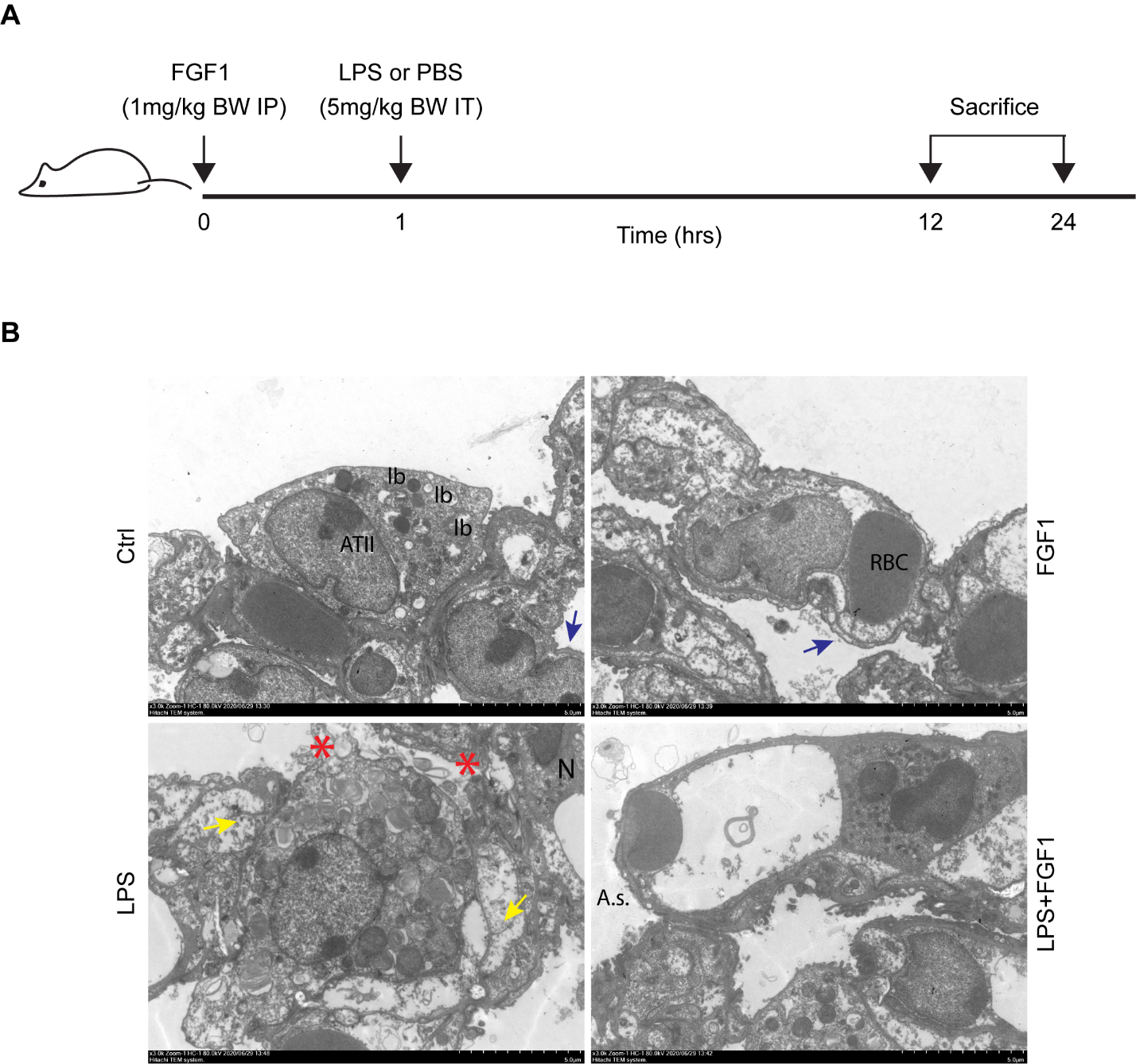


**Additional file 1: Figure S1.** (A) Schematic of the experimental procedure with a timeline. (B) Representative transmission electron micrographs showing ultrastructural changes in LPS-induced ALI, taken at 12 hrs. The scale bar is 5µm. Blue arrows indicated alveolar-capillary barrier, yellow arrows indicated edema and thickened septa containing infiltrates, red asterisk indicated disrupted endothelial-capillary barrier. Mean (SEM), n=5 per group. ATII denoted alveolar type II cells; lb, lamella bodies; RBC, red blood cell; N, neutrophil; A.s, alveolar space, respectively.
