# Supplementary material for: FGF1 alleviates LPS-induced acute lung injury via suppression of inflammation and oxidative stress
Source: Mol Med. 2022 Jun 28;28:73. doi: 10.1186/s10020-022-00502-8 (PMC9238076; doi:10.1186/s10020-022-00502-8)
Supplement: Supplementary file 2 — Additional file 2: Figure S2. (A) Western blot analysis and quantification of the relative protein expressions of antioxidant enzymes Catalase and GPX4 normalized to GAPDH. Data are presented as mean ± SD (n = 5 per group). *p < 0.05; One-way ANOVA with Tukey’s post-hoc test. [file 10020_2022_502_MOESM2_ESM.docx]

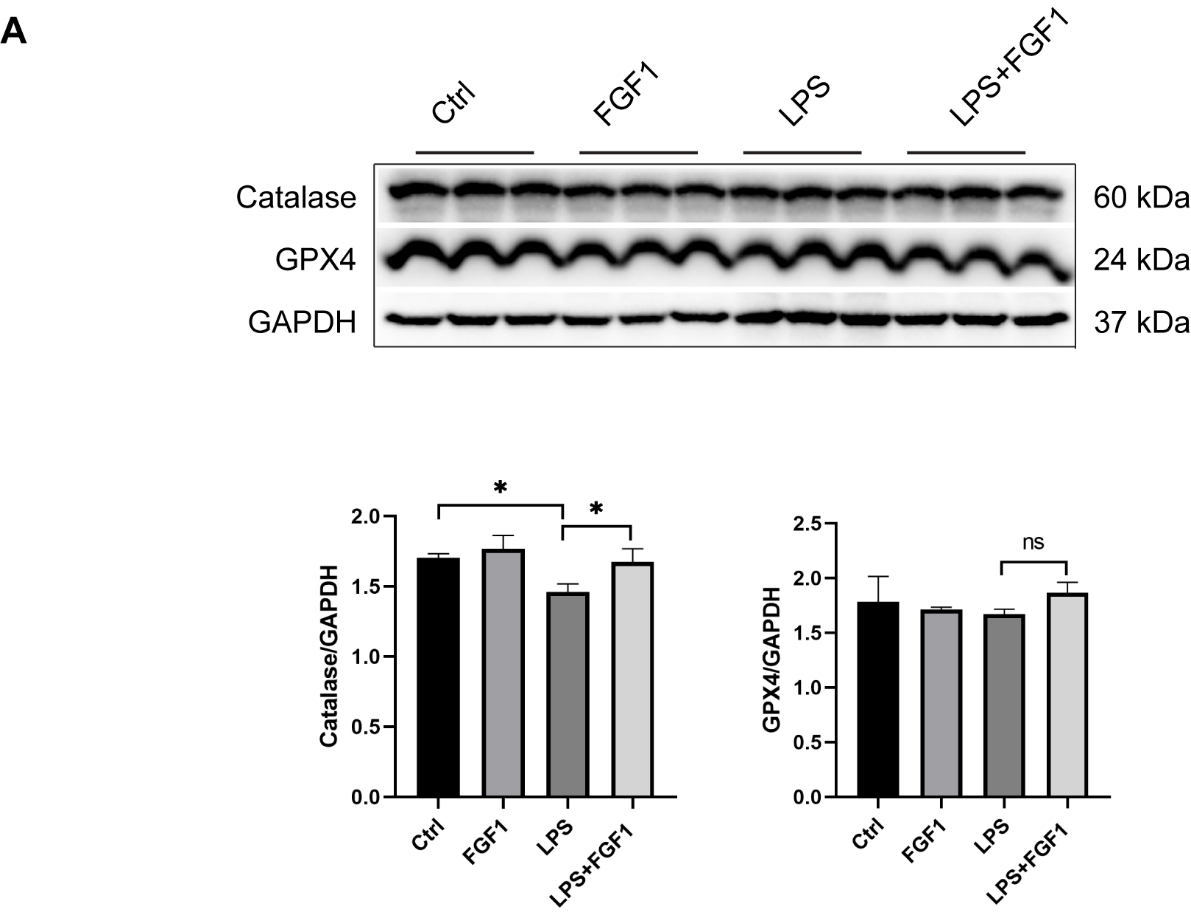


**Additional file 2: Figure S2.** (A) Western blot analysis and quantification of the relative protein expression of antioxidant enzymes Catalase and GPX4 normalized to GAPDH. Data are presented as mean ±SD (n=5 per group). *p < 0.05; One-way ANOVA with Tukey’s post-hoc test.
